# Pathophysiology of Postoperative Hearing Disorders after Vestibular Schwannoma Resection: Insights from Auditory Brainstem Response and Otoacoustic Emissions

**DOI:** 10.3390/jcm13071927

**Published:** 2024-03-27

**Authors:** Idir Djennaoui, Mathilde Puechmaille, Chloé Trillat, Justine Bécaud, Nicolas Saroul, Toufic Khalil, Paul Avan, Thierry Mom

**Affiliations:** 1 Department of Otolaryngology Head Neck Surgery, University Hospital Center of Hautepierre, 1 Avenue Moliere, 67000 Strasbourg, France; idir.djennaoui@chru-strasbourg.fr; 2 Department of Otolaryngology Head Neck Surgery, University Hospital Center, Hospital Gabriel Montpied, 58, Rue Montalembert, 63000 Clermont-Ferrand, France; mpuechmaille@chu-clermontferrand.fr (M.P.); ctrillat@chu-clermontferrand.fr (C.T.); jbecaud@chu-clermontferrand.fr (J.B.); nsaroul@chu-clermontferrand.fr (N.S.); 3 Department of Neurosurgery, University Hospital Center, Hospital Gabriel Montpied, 58, Rue Montalembert, 63000 Clermont-Ferrand, France; tkhalil@chu-clermontferrand.fr; 4 Department of Biophysics, School of Medicine, University of Clermont Auvergne (UCA), 63000 Clermont-Ferrand, France; paul.avan@uca.fr; 5 Mixt Unit of Research (UMR) 1107, National Institute of Health and Medical Research (INSERM), University of Clermont Auvergne (UCA), 63000 Clermont-Ferrand, France

**Keywords:** vestibular schwannoma, hearing preservation, otoacoustic emissions, auditory brainstem response, retrosigmoid approach

## Abstract

**Background**: In order to better understand the pathophysiology of surgically induced hearing loss after vestibular schwannoma (VS) surgery, we postoperatively analyzed the hearing status in a series of patients where hearing was at least partially preserved. **Methods**: Hearing was assessed through tonal audiometry, speech discrimination score, maximum word recognition score (dissyllabic word lists—MaxIS), otoacoustic emissions (OAEs), and auditory brainstem response (ABR). The magnetic resonance imaging (MRI) tumor characterization was also noted. **Results**: In a series of 24 patients operated on for VS over 5 years, depending on the results of this triple hearing exploration, we could identify, after surgery, patients with either a myelin alteration or partial damage to the acoustic fibers, others with a likely partial cochlear ischemia, and some with partial cochlear nerve ischemia. One case with persisting OAEs and no preoperative ABR recovered hearing and ABR after surgery. Long follow-up (73 ± 57 months) revealed a mean hearing loss of 30 ± 20 dB with a drastic drop of MaxIS. MRI revealed only 25% of fundus invasion. **Conclusion**: a precise analysis of hearing function, not only with classic audiometry but also with ABR and OEAs, allows for a better understanding of hearing damage in VS surgery.

## 1. Introduction

Nowadays, resection of vestibular schwannoma (VS) not only aims at achieving total or near-total tumor removal, but also at sparing facial function and, if possible, auditory function. In actuality, a vast majority of VSs are benign tumors, and only become life-threatening in case of brainstem compression and/or elevation of intracranial pressure. 

Therefore, functional impairment has become less and less acceptable, as a therapeutic sequel in mostly asymptomatic patients, with no brainstem compression. Monitoring of facial function is routinely used with success, leading to a very low rate of postoperative facial nerve palsy [1]. Hearing preservation, by contrast, remains a hard task, even though several techniques of auditory monitoring have been used for many years, e.g., auditory brainstem response (ABR) [2], electrocochleography (EcoG) [3], and DPOAEs [4], with a rate of auditory preservation hardly reaching 50% of cases in most teams [5,6,7,8,9].

In order to improve our rate of hearing preservation, especially since an increasing number of patients present, preoperatively, with nearly normal hearing or a small hearing loss [10], a better understanding of the deleterious mechanisms occurring during surgery is mandatory.

Herein we have postoperatively sorted out our patients through their auditory pattern, using pure tone audiometry, speech discrimination, and electroacoustic exploration with ABR and transiently evoked otoacoustic emissions (TOAEs) or distortion-product otoacoustic emissions (DPOAEs). By checking the electroacoustic exploration for each case, and comparing it to the auditory status, we attempted to clarify the pathophysiology leading to postoperative hearing loss.

The rationale was that, depending on the deleterious mechanism to the auditory function, the pattern of electroacoustic exploration would differ. TOAEs and DPOAEs reflecting the functional status of the cochlea, combined analysis of ABR, TOAEs/ DPOAEs, and audiometry can reveal the damaged site along the auditory pathway, from the cochlea to the brainstem.

This series finally helped us better understand the pathophysiology of hearing alteration in patients operated on for VS and we accordingly modified our surgical technique in the aim of eventually better preserving hearing.

## 2. Materials and Methods

This retrospective study was conducted by the analysis of charts of patients who had preserved hearing after a conservative surgical procedure for VS, operated on between 2009 and 2014. Since 2014, the vast majority of small VS, that is, ≤stage 2 (KOOS classification), are either scanned or treated by radiosurgery in our institution. That is why this retrospective series did not consider cases operated on after 2014.

Most cases were ≥stage 2 tumors. In the rare big tumors (stage ≥ 3), a near-total or subtotal tumor removal was achieved, in order to preserve facial function first, then the auditory function when possible.

All patients included had a good preoperative hearing performance, with a grading score between A and B (grading system of the American Academy of Otolaryngology—Head Neck surgery, AAO-HNS). One case with poor preoperative hearing was included because there was a hearing recovery after surgery.

The surgical procedure used was a retrosigmoid approach with, in some cases, hearing monitoring through DPOAEs or cochlear microphonics (CM) through EcoG, with some cases included in previous studies [3,4] but not direct VIIIth nerve action potential recording. When intraoperative CM was monitored, we used the Echodia system (Clermont-Ferrand, Lyon, France) with a soft and malleable golden-coated probe inserted within the external ear canal. In all cases, both an otolaryngology (ENT) team and a neurosurgical team operated on patients together. The neurosurgical team would achieve the retrosigmoid approach, then debulk the tumor and leave place to the ENT team once the tumor had been shrunk to a stage 2-like tumor. The ENT team would then open the internal auditory meatus (IMA), and dissect the tumor from the surrounding nerves, that is, facial and, if possible, cochlear nerves, removing the tumor backwards towards the cerebellopontine angle (CPA) with the help of continuous irrigation through specifically made surgical tools (Prof. Mom’s tools, Integra^®^-MicroFrance^®^, Saint-Aubin-Le-Monial, France).

Although, the hearing preservation was considered as useful on the AAO-HNS grading score between A and B, the postoperative hearing was considered, in this study, as serviceable when the speech discrimination score (SDS) was >50% with monosyllabic word lists at 35 dB HL above the intensity of the intelligibility threshold (giving 50% of understood words), or the maximum intelligibility score (MaxIS) ≥ 70% in disyllabic word lists.

All patients involved in this study had a tonal and speech audiometry one to three months after surgery in our department, with some of them undergoing an additional audiometric test several years after when the records of follow-up were available.

For each case, the audiometric criteria reported were the MaxIS, the pure tone average (PTA) calculated by averaging 0.5, 1, 2, and 4 kHz hearing thresholds, and the SDS.

The last two parameters allowed us to classify those cases according to the AAO-HNS classification. In the case of patients with good hearing preservation but with hearing complaint, we looked for auditory fatigue by a PTA before and after 2 min of noise exposure (80 dB HL) and by checking ABR waves in function of duration of sound stimulation (carried out at 90 dB HL).

All patients had ABR 1 to 6 months after surgery. ABRs were recorded using an electrode placed on the mastoid and referred to the vertex. Measurements were conducted in a sound-proof room. Click stimuli were presented at 90 dB HL through an ear receiver at 19 Hz with contralateral masking. Click phases were alternately reversed. The responses to 1000 stimuli were filtered with a band pass filter of 50–3000 Hz, then amplified and averaged using a signal processor using either ECHODIA Elios (Clermont Ferrand, Lyon, France) or the Interacoustics ECLIPSE device (Middlefart, Denmark).

After averaging 1000 clicks, the analysis was made by the same examiner who first sorted out patients with identified I, III, and V waves, whatever their latency (“Good ABR”), from the others with no waves or only an identified wave V. Then, the latter patients were grouped into two categories:

- Presence of only wave V: ABRs were considered as impaired but still present.
- Absence of any identifiable wave: ABRs were considered as absent.

Because most patients had preoperative increased ABR latencies, we did not consider this ABR characteristic, but only the alteration of ABR waves.

Most patients had preoperative recordings of TEOAEs. When present, these were, in most cases, checked postoperatively using the classical device from Kemp, ILO88, or the ECHODIA system. A ≥50% reproduction was considered normal.

Based on preoperative MRI, we classified the tumor according to KOOS classification. We also defined the VS location considering its penetration in the IMA; that is, with a small penetration in the medial third (named “porus”), or with at least a full filling of the medial third, leaving the lateral third free (medial third), or with a penetration up to the lateral third of the IMA, and in the latter case, reaching or not the modiolus of the cochlea.

All patients hospitalized in our University hospital were clearly informed that their charts could be anonymously used for medical publication and that they were free to refuse so, and there were no refusals. This retrospective study was approved by local ethics committee (IRB00013412, “CHU de Clermont Ferrand IRB #1”, IRB number 2023-CF118) with compliance to the French policy of individual data protection.

## 3. Results

During this period, 30 patients were operated on with an attempt of hearing preservation, with 24 (80%) cases with a certain amount of hearing preservation, but only 10 (33.3%) with a full hearing preservation.

The auditory monitoring was helpful when any drop in signal was spotted, leading us to stop any surgical maneuver and to gently irrigate the operative field with cool saline, waiting for recovery

### Postoperative Auditory Status

Of the 24 cases assessed, the mean age of the operated patients was 50 ± 12 years old (nine women).

The early postoperative MaxIS score was at 92 ± 14% [50–100%].

The average intelligibility threshold was 39 ± 9 dB [10–65 dB]

The average SDS (n = 18, 6 SDS missing) was at 85 ± 19% [50–100%],

The audiometric curve shapes were disparate. We sorted out several types of postoperative pure-tone audiometry (PTA):

- A global preserved (type GP) audiometry with ≤15 dB loss.
- A sharp hearing loss (type Sharp HL) in higher frequencies from 1 kHz or higher frequencies with preserved lower frequencies.
- An altered audiometry, but still with a useful hearing (at least AAO-HNS grade C).

Cases were sorted out according to the results deducted from their audiologic exploration (Table 1)

**Figure 1:**
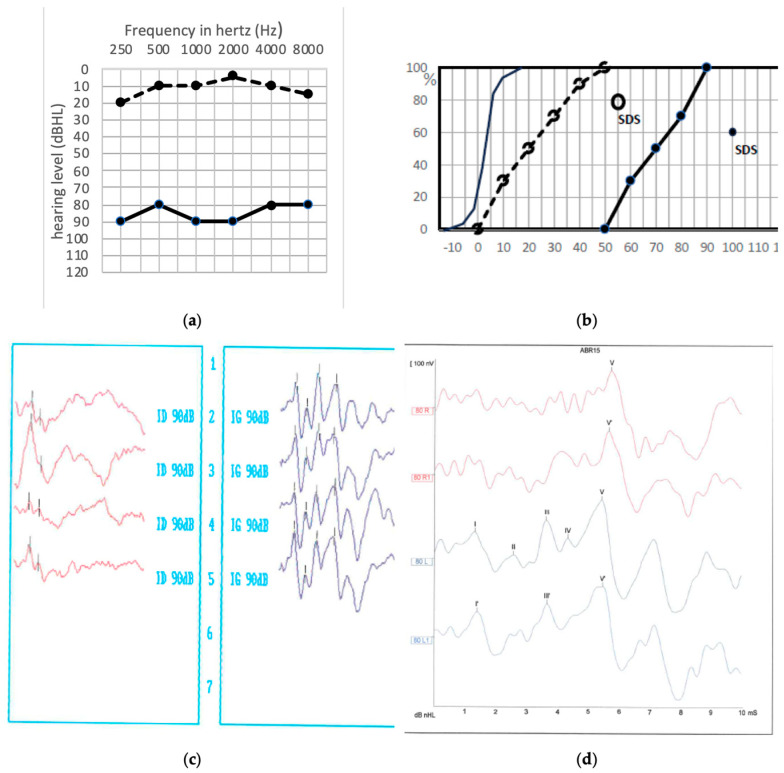
(**a**) Pre- and postoperative audiogram of the patient in whom a hearing improvement was noted (bone conduction not shown). Solid line: preoperative hearing levels, dotted line: postoperative hearing levels. (**b**) pre- and postoperative speech audiometry in silence with dissyllabic word lists. The speech discrimination score, SDS, was recorded with monosyllabic word lists. Preoperative SDS was not recorded because the required intensity was >100 dB HL. Solid points: preoperative hearing levels, empty points: postoperative hearing levels. (**c**,**d**) ABR recording before and after surgery. (**c**): preoperative situation, red column: impaired right side, blue column: safe left side. (**d**): postoperative situation where the wave V is clearly identified on both sides (**Right side**: red curves, **Left side**: blue curves).

Lastly, in only one case, the patient recovered from his preoperative hearing loss after surgery, the PTA improving form 83 dB HL preoperatively to 10 dB HL postoperatively (Figure 1a,b). Interestingly, in this case, ABR was absent in the preoperative functional exploration, while after surgery, with the PTA recovery, ABR reappeared, although with delayed latency (Figure 1c). Preoperative TOAEs were present and persisted after surgery.

**Figure 2:**
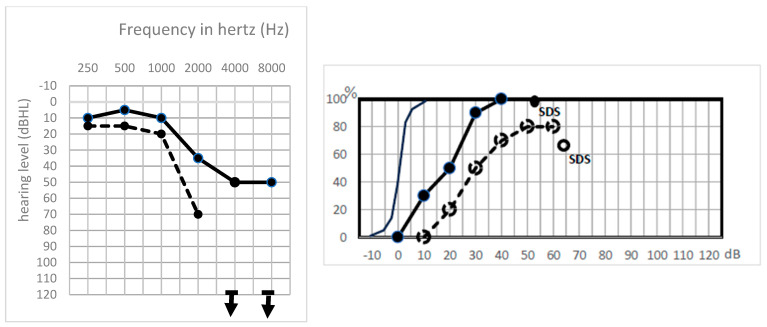
Preoperative (solid line) and postoperative (dashed line) tonal and speech audiometry of a patient with a sharp amputation on high frequency thresholds. Solid line: preoperative audiogram, dotted line: postoperative audiogram. Solid points: preoperative hearing levels, empty points: postoperative hearing levels. Downward arrows: hearing level is beyond 120 dBHL.

Type Sharp HL was a frequent situation (six cases, 25%). It is characterized by a sharp threshold decrease on high frequencies (>1 kHz) (Figure 2).

These cases with good lower frequencies and presence of TOAEs had a good cochlea, while the absence of ABR associated with no high frequency perception proved the severing of higher-frequency acoustic fibers while the cochlea was still intact.

**Figure 3:**
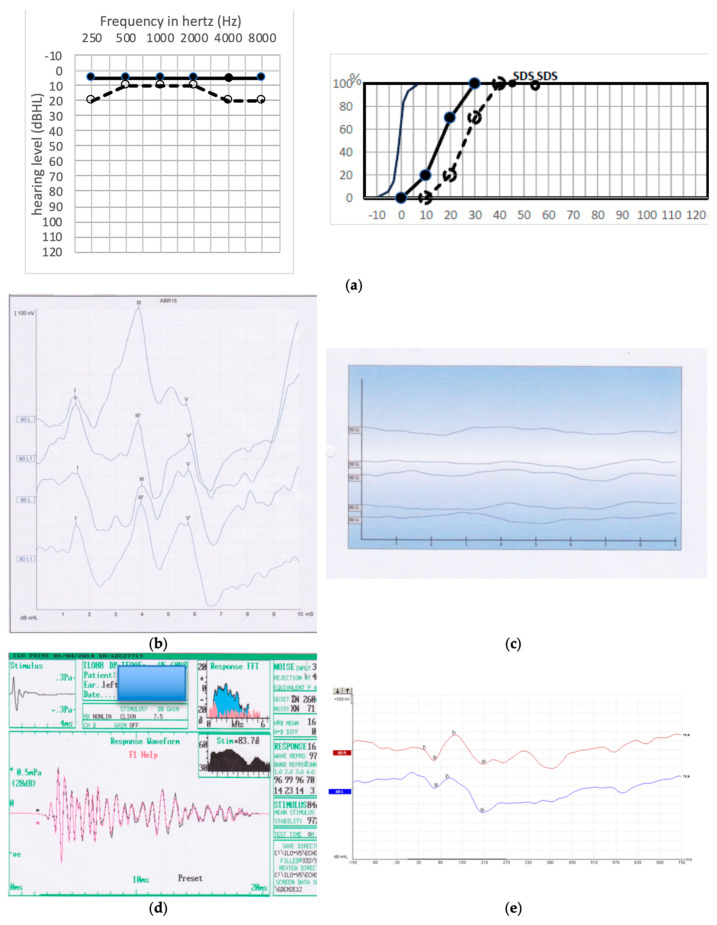
(**a**) Tonal and speech audiometry for patient 10 (GP 1). Solid line: preoperative audiometry, dashed line: postoperative audiometry. (**b**) Preoperative left ABR recording in patient 10, waves I, III V are clearly identified, waves with prime symbols correspond to an additional measure. (**c**) Postoperative left ABR recordings in patient 10, showing no response. (**d**) Postoperative TOAEs in patient 10 (GP 1). (**e**) Postoperative recording of auditory cortically evoked responses for the same patient 10 (GP 1): the N_1_, P_1_, and N_2_ waves are clearly identifiable, upper red curve: right ear, lower blue curve: operated left ear.

The two cases of GP 1 type had a very good hearing with TOAEs but no ABR (Figure 3b–d). The speech intelligibility was very good, showing that cortical integration was achieved. In one case (patient 10), we tried to collect a cortically evoked response, which proved to be present (Figure 3a–e).

In these two GP 1 cases, the absence of ABRs indicates a damage to the cochlear nerve. However, because speech intelligibility was good, and that, at least in one case, we had normal cortical ABR, we were able to deduce that the cochlear nerve was still able to conduct the auditory neural potentials to the cortex. A likely explanation is that the myelin sheet had been altered during the surgical procedure in the CPA, while the acoustic fibers were not severed. The alteration of the myelin sheet after healing could preclude ABR identification by a likely dysynchrony of the neural potential speed conduction between acoustic fibers. We named this group “Myelinopathy”.

**Figure 4:**
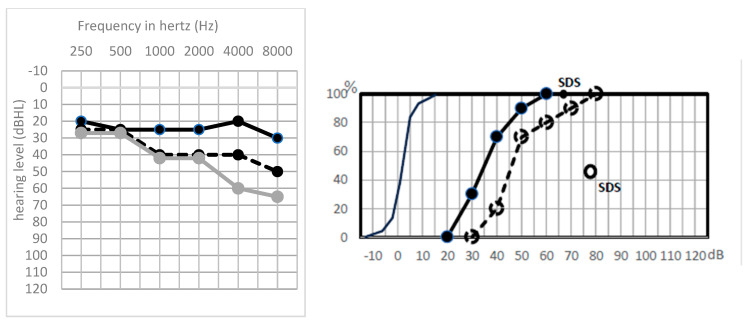
Tonal and vocal audiogram illustrating hearing fatigability phenomenon, solid line: before surgery, dotted line: after surgery, grey line: after 80 dB sound exposure for two minutes.

In the two cases of GP 2 subtype, ABRs were fluctuating and tiring, clearly appearing during the first 200 or 300 stimulations and vanishing as the averaging would progress. This phenomenon, thus, pointed to a wounded, but not severed, cochlear nerve These cases were associated with an auditory fatigue that came out with increasing thresholds on higher frequencies after exposure to an 80 dB HL white noise for 2 min (Figure 4). One case had good TOAEs, indicating the cochlea was healthy. The other one had no TOAEs, indicating that the cochlea also, together with the cochlear nerve, was suffering, while still working. In the two GP 2 cases, ABR exhibited only wave V, even with a supraliminar acoustic stimulation.

One can think that the auditory fatigue in GP 2 is underlined by a functional alteration of the nerve. We named this group “Neuropathy”.

**Figure 5:**
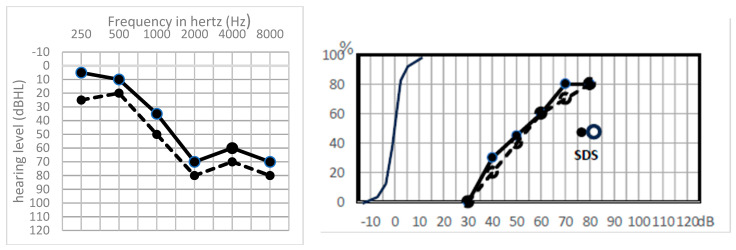
Tonal and vocal audiogram of a patient in subgroup GP 3. Solid line: before surgery, dotted line: after surgery.

Most patients of GP 3 type (Figure 5) had postoperative hearing that was almost unchanged (<20 dB loss), with no signs of auditory fatigue, likely indicating that the surgery did not induce damage to the cochlea or to the acoustic fibers.

**Figure 6:**
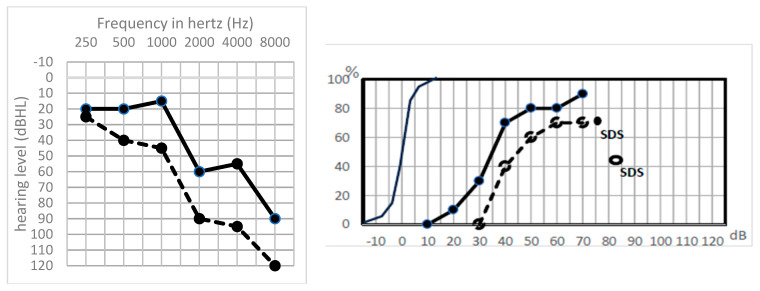
Tonal and speech audiogram of a patient in subgroup GP4. Solid line: before surgery, dotted line after surgery.

In the last group, GP 4 type (Figure 6), the loss of TOAEs was associated with good ABR and hearing preservation, proving that, although altered, the cochlea could work enough to give birth to ABR through a still-functional cochlear nerve. A partial cochlear ischemia is highly suspected in this group, which could be responsible for a drop of the endolymphatic potential and could account for a loss of DPOAEs with a still-working organ of Corti [11].

According to AAOHNS classification, hearing performances were considered as useful (hearing class A and B) in 17 cases (71%), altered but still serviceable (class C) in 6 cases (25%), with one case in class D who was happy with a good MaxIS, despite a poor SDS.

In 18 cases, a delayed hearing control could be identified (mean follow-up: 73 ± 57 months [6 months–17 years]. A global hearing loss of 30 ± 20 dB, on average, was observed with a drastic MaxIS alteration of 45 ± 25 dB. SDS was not available. Only two patients kept a MaxIS ≥ 70%.

When scrutinizing differences between groups, we could observe the following patterns (Table 2):

MRI information: There were seven tumors (29.16%) located at the fundus, with four of them reaching the modiolus; six cases (25%) with invasion of the medial third; ten cases (41.66%) located at the porus; and the last one purely intracranial with no contact with the porus.

There was no correlation between the auditory profiles and the MRI location. The case with full recovery was the purely intracranial one.

## 4. Discussion

Hearing can be harmed during all surgical steps of cerebellopontine angle (CPA) tumor removal once the dura mater is crossed. The critical stages are essentially coagulation near the cochlear nerve, removal of the intrameatal portion of the schwannoma, and drilling of the internal auditory canal [12].

Very few reports in this field are available to date in the literature. We found a recent series from Sass et al. [13] in 2019, who reported hearing preservation attempts in 31 cases; the previous series being older, although informative.

Apart from total hearing loss cases, pathophysiology of postoperative partial hearing loss is poorly understood. Herein, we were able to elucidate four different types of hearing deleterious events thorough auditory exploration, which are partial nerve severing, surgically induced myelin disorder, transient suffering of the acoustic fibers, and partial cochlear ischemia, which were not identified to date to our knowledge, except from cochlear ischemia [4,12]. It could also be likely that neural edema or swelling could account for some degrees of, at least transient, hearing loss, but we had no means to prove it in this series.

The cochlear nerve is particularly fragile at its CPA portion (8–10 mm from its emergence). In this area, nerve fibers are recovered by oligodendrocytes, which, unlike Schwann cells located more peripherally, are devoid of two glycoproteins, the Myelin-associated glycoprotein and Po glycoprotein [14,15], whose role is to strengthen the compactness of myelin. This feature makes the nerve more sensitive to mechanical and thermal traumas, compared to other cranial nerves, which are covered by a central myelin type on only a few millimeters [16,17].

Also, the cochlear blood supply, provided by one or two tiny labyrinthine arteries, is at high risk during CPA tumor surgery.

In order to sort out the deleterious mechanisms of hearing loss in CPA surgery, we explored the auditory function, not only through audiometry, but also through ABR and TEOAEs.

In actuality, we know that TEOAEs are very sensitive to cochlear damage, and in particular to cochlear ischemia [4]. OAEs have been used for hearing monitoring during CPA tumor removal [4,18] and are underlined by strong experimental data in small rodents [14,19,20,21]. The cochlear function can also be intraoperatively probed through electrocochleography (EcoG); in particular, the cochlear microphonics (CM) [3] or cochlear nerve action (CNAP) potential [22,23,24]

It has to be recalled that some authors reported the absence of parallelism between hearing results and the cochlear electrophysiological recordings. Silverstein et al. [25] reported the persistence, during removal of the tumor, of an EcoG signal twenty-five minutes after the severing of the auditory nerve.

These findings have also been demonstrated experimentally by Rosahl et al. [26], who found the presence of short-latency electrical potentials after transection of the cochlear nerve in the CPA, thus confirming that the control of integrity of the auditory nerve should not only rely on EcoG.

ABRs have been widely used for the diagnosis and auditory monitoring of patients with CPA tumors; these are electrical potentials collected in a distant field, by an acoustic stimulation—generally by clicks—and averaged. This stimulation “constrains” the fibers of the cochlear nerve to synchronously trigger a nervous impulse, which entails the appearance of several waves, each wave representing the activation of a specific generator along the auditory path, which extends from the cochlea to the brainstem.

The relevance of intra- and postoperative ABRs have been the subject of several controversies. Some authors rather evoke the good adequacy between the good shape of ABR and the postoperative auditory results [27].

For many clinical neurophysiologists, the wave V is considered to be the best electrophysiological indicator of damage to the auditory nerve during surgery [28,29,30], although, Matthies and Samii [2] emphasize the role of wave III.

Also, some studies have shown that absent ABR waveforms have not been a negative prognostic sign regarding hearing preservation, notably the absence of wave V [31]. In one report, hearing preservation was achieved without detectable wave V in 37.5% [32].

In our series of hearing preservation with cases of partial hearing preservation, we were able to distinguish four groups of patients with postoperative preserved hearing, based on their postoperative PTA and discrimination capacity, together with their ABR and TOAE patterns. The rationale is that TEOAEs could provide reliable information on the cochlear function, while ABRs could provide good information on the cochlear nerve status. One remarkable fact is that ABRs can be totally disorganized or absent with, nevertheless, a preserved and useful postoperative hearing function, as in the two cases of GP 1.

The absence of ABRs despite good hearing results demonstrates the persistence of the hearing information flow through auditory pathways up to the auditory cortex. Indeed, speech intelligibility in the early postoperative period was excellent in GP 1 cases, while there were no ABRs. We had the opportunity to record cortically evoked auditory potentials (Corticals) in one GP 1 case that were undoubtedly present despite the absence of ABRs (Figure 3a–e). Further, the intraoperative loss of ABR is known to be poorly informative, thereby making the prognostic value of CNAP significantly higher than that of ABR [24,33].

Because in GP 1 cases there was no PTA deterioration, we were able to deduce that the absence of ABR despite good hearing is due to cochlear nerve myelin sheet alteration. It could be a kind of surgically induced myelin scar precluding any good synchrony of auditory fibers in the CPA, thus resulting in no ABR. Because the Corticals were recorded and the speech intelligibility was good in GP 1, one could say that the auditory nerve potentials eventually resynchronized when reaching the cortex. Desynchronization is defined by the loss of spike timing between neurons, resulting in severe effects on the wave formation of the ABRs, which is the sum of the synchronized spike activities of the neurons evoked by sound stimuli [34]. Such neural dysynchrony has been reported on several retrocochlear-alteration-inducing auditory neuropathies, such as Friedreich’s ataxia, spinocerebellar degeneration, and multiple sclerosis [35,36,37,38,39,40,41,42,43]. This pattern could account for the two GP 1 patients.

In the Sharp HL group, we found that hearing was completely lost from medium to higher frequencies. The mechanisms that seem most relevant to explain the loss of ABR, especially when TEOAEs were still present, is the acoustic nerve being partially severed at the fibers carrying the high frequencies, which are thought to be the more superficial at the cochlear nerve [44]. Because ABR mainly relies on fibers coding from 2–4 kHz [45,46], it is not surprising that ABR vanished after surgery in the Sharp HL group. These patients had a pretty good MaxIS, despite the loss of higher frequencies, likely due to the perception of part of the dissyllabic words. They were happy with their hearing without hearing aids for years. The TEOAE profiles are related to the degree of hearing impairment—their presence requires hearing thresholds lower than 30 dB on medium and low frequencies. They were detectable in two out of six cases with mild hearing loss (<30 dB), in two cases they were already absent preoperatively and in two cases absent probably due to other interrelated pathophysiological mechanisms. Those patients could maybe benefit from frequency-transposition-processing hearing aids [47]. However, none of those in our series would accept this type of hearing rehabilitation.

An auditory fatigue was revealed in the two GP 2 patients. The wave V could be spotted on ABR, although delayed, while the others were not identified. In addition, while wave V could be identified for sure after 300–500 averages of 90 dB HL sound stimuli, it tended to disappear when the averaging was too long, at 1000 stimuli. This auditory nerve fatigue is known in some patients, such as in cases of Friedreich ataxia [48]. Herein, we also were able to reveal the auditory fatigue in audiometric tests in noise. It is likely that the nerve had suffered from surgical dissection. The deleterious mechanism remains obscure, but the acoustic fibers kept on functioning with good PTA and identifiable wave V. This auditory profile may be due to a partial cochlear nerve ischemia or to a postoperative swelling of the nerve, compromising but not precluding its global nerve function. The deterioration of the ABR could also correspond to a type of auditory neuropathy induced by a synaptic abnormality between the inner hair cells and the afferent auditory neurons [43,49,50]. The study performed by El Badry et al. [51] on the attempt to make an animal model of auditory neuropathy by treating chinchillas with carboplatin, thus destroying a large part of inner hair cells and auditory nerve fibers, showed that the ABR, as well as hearing thresholds, remained only slightly modified, indicating that the synchrony of the remaining neurons had not been affected. Thus, it suggests that the dyssynchronization of the neural response during auditory neuropathy could result from a different mechanism than the loss of inner hair cells and auditory nerve fibers.

Cochlear ischemia is likely involved in sensorineural hearing loss during CPA surgery; apart from arterial section that leads to total hearing loss, any surgical manipulation in the CPA could induce labyrinthine artery (LA) vasospasm [15]. The occurrence of long-lasting vasospasm of the LA with reversible loss of cochlear function has been demonstrated in a rabbit model, whereby cochlear blood flow (CBF) and hearing function were simultaneously monitored during surgical manipulation of the VIIIth nerve bundle in the CPA. In particular, it was shown in this model that DPOAEs had a high capacity of tracking any alteration of CBF, reacting in about 10 s to cochlear ischemia and even faster, in less than 5 s, to CBF recovery.

Morawski et al. [52] reported that tumor debulking, cauterization near the internal auditory canal, and stretching of its contents were able to affect DPOAEs, sometimes irreversibly. Interestingly, they observed that the most specific pattern of DPOAE change portending postoperative hearing loss was that of a rapid loss of DPOAEs at all frequencies. Mom et al. [4] have shown that apart from a steady DPOAE profile where postoperative hearing remained good, a transient DPOAE profile with oscillating responses corresponding to successive series of reversible vasoconstrictions of LA led to mild hearing loss. CM proved also to be, intraoperatively, very informative in CPA surgery and could also detect vasospasm [3].

Within this series, such pattern would be attributed postoperatively to GP 4, where ischemia phenomenon would alter outer hair cell function and mechano-transduction, leading to postoperative loss of TEOAEs, but with preservation of ABRs when acoustic stimulation was carried out at 90 dB HL, triggering a nervous response with well-identified waves. This could be responsible for a moderate hearing loss, even greater in two cases in the short postoperative period, that recovered a few months later.

In the same reasoning, the spectacular case with hearing restoration after surgery, in which TOAEs were always recorded in the pre- and postoperative period, could thus not be explained by cochlear ischemia. The preoperative absence of ABR that recovered after surgery likely shows that the neural pathway was released by surgical removal.

In this report, we had the chance to perform auditory exploration many years later. We would expect hearing status to be pretty unchanged in time. In actuality, in most cases, hearing deteriorated drastically, in particular the speech intelligibility, as seen through the MaxIS, that dizzyingly dropped down. Finally, only one case had a MaxIS ≥ 70%, with 11/18 cases with a MaxIS ≤ 50%. We can guess that most of these patients would have been AAO-HNS class D if the SDS (recorded with monosyllabic word lists, thus always inferior to MaxIS) had been recorded.

Looking at the late hearing performance was interesting, showing different evolutions of hearing between groups.

In the Sharp HL group, with time, the loss of hearing located at the higher frequencies extended towards the lower frequencies. This can explain the MaxIS deterioration. We have no evidence to explain this hearing worsening to the lower frequencies. It could be a progressive alteration of the adjacent acoustic fibers.

In GP 1 patients, with proven myelin alteration, we expected a good hearing preservation, since hearing performance was excellent in the early postoperative period. But we observed a progressive hearing deterioration over the years. Here again, we do not know which deleterious mechanism finally deteriorated hearing. One can think that the “myelin scar” progressively altered the acoustic fiber’s function.

In the two GP 2 patients, where we expected an eventual complete deafness with time, we were surprised to observe a good PTA preservation in the two cases. One of them had the best MaxIS score of the series at 70%. But the other one deteriorated drastically in terms of his intelligibility, the MaxIS passing from 100% to 35%.

When the postoperative PTA was well-preserved, in GP 3, again, we expected a good late hearing preservation, but it was not the case within this group, who also saw global deterioration of MaxIS, although the PTA was rather well-preserved. Despite the apparent unarmed procedure, the cochlea and auditory pathway did suffer from the CPA surgery.

Hearing aids could give good results in these cases of postoperative partial hearing preservation. That being said, we know that the cochlea remains very vulnerable during the recovery period, in particular to sound exposure, in case of cochlear reversible ischemia [53]. Most patients of this series did not use any hearing aid after surgery, and yet hearing deteriorated. This proves that the cochlea, together with the cochlear nerve, can be impacted by surgery to the CPA, even though early postoperative hearing has been preserved.

Previous studies have reported a hearing deterioration with time when VSs are only scanned, with about 55% of cases retaining their hearing at 10 years (Class A or B AAO-HNS) [54]. It is also known that radiosurgery is responsible for an important rate of hearing deterioration over time—a systematic review found that hearing preservation rate was 58% at an average reporting time of 46.6 months after radiotherapy treatment, but analysis of time-based reporting shows a clear trend of decreased hearing preservation extending to 10-year follow-up [55]. Our surgical series also shows a trend of hearing deterioration with time, especially in terms of speech intelligibility, as reflected by the MaxIS, with only one out of eighteen cases with a MaxIS ≥ 70%, despite a mean PTA around 30 dB on average.

However, from our standpoint, it is always a good thing to preserve hearing. In our series, most patients were happy with their residual hearing for years. Maybe preventive treatment with antioxidants could prevent the drastic hearing deterioration in the long term, but this remains to be proven.

This auditory classification, based on a putative pathophysiology, could hopefully help improve the hearing preservation rate by leading to a better understanding of the alteration of electroacoustic signals used for intraoperative hearing monitoring.

Based on our data, we now stop any dissection procedure when the cochlear signal (DPOAEs or CM) drops and cool the operative field with saline, waiting for its recovery; we use a sharp dissection in the tumoral capsule close to the nontumoral nerves, but not against these nerves.

With regard to MRI findings, regardless of tumor volume, there was no correlation with the auditory profile and fundal invasion, which is in line with the literature findings in the preoperative period [56,57]. On the other hand, the invasion of the most lateral part [58,59,60] of the IAC or the cochlear fossa [61] is a predictive factor of postoperative hearing impairment. Most teams would report that the best chance of hearing preservation when surgery is decided is for small tumors < 1 cm [62,63], which we no longer operate on in our institution.

## 5. Conclusions

We have shown four different deleterious mechanisms to hearing function through the thorough analysis of hearing in a series of partial hearing preservation. In addition to cochlear ischemia, which can compromise hearing during surgery to the CPA, we have evidenced that higher-frequency fibers are at risk of being wounded, together with the myelin sheet of the cochlear nerve. The functional alteration of the nerve without severing can also occur. The progressive deterioration of hearing function, despite early good performance, remains mysterious and requires basic research on animal models to be explained.

## Figures and Tables

**Table 1:** Table representing the distribution of patients according to the results deduced from the audiometric and electrophysiological profile.

n = 24	PTA Type	TOAEs	ABRs	Hypothesis
Sharp HL (n = 6)	Total higher hearing frequency loss	Good or absent	Absent	Partial nerve severing
GP 1 (n = 2)	Flat preserved audiogram	Good	Absent	“Myelinopathy”
(GP 2) (n = 2)	Flat preserved audiogram	Good or absent	Increased latencies with auditory fatigue	“Neuropathy”
GP 3 (n = 10)	Flat preserved audiogram	Unchanged	Unchanged	Full preservation
GP 4 (n = 4)	Hearing loss in all frequencies	Absent	Good	Partial cochlear ischemia

**Table 2:** Auditory characteristics of patients followed-up in the long term (mean follow-up: 75.7 ± 56.8 months).

Initial Group	Distant PTA Pattern	Individual Raw PTA Loss (dB)	Individual Initial Postop MaxIS	Individual Late Postop MaxIS
Sharp HL n = 4	Total higher hearing frequency loss extending to the lower frequencies	45/60/30/ 40	100/90/90/ 80	65/60/65/ 50
GP 1 n = 2	Global hearing loss in all frequencies	40/60	100/100	50/0
GP 2 n = 2	unchanged	15/ 20	100/100	35/70
GP 3 n = 7	Unchanged or loss mainly on higher frequencies	0/0/10/ 60/20/20/20	70/100/100/ 100/70/100/100	60/10/35/ 0/55/25/35
GP 4 n = 3	Hearing loss mainly in high frequencies	40/40/15	100/100/80/ 80	50/80/35

## Data Availability

Our data are stored confidentially in our institution and protected for ethical reasons.
